# A Tricky Diagnosis of Poncet’s Disease

**DOI:** 10.7759/cureus.32972

**Published:** 2022-12-26

**Authors:** Maria Helena Lourenço, Ana B Silva, Jaime C Branco, Inês Silva

**Affiliations:** 1 Rheumatology, Egas Moniz Hospital, Centro Hospitalar de Lisboa Ocidental, Lisbon, PRT; 2 Comprehensive Health Research Center (CHRC), NOVA Medical School, Lisbon, PRT; 3 Faculty of Medicine, NOVA Medical School, Lisbon, PRT

**Keywords:** poncet's disease, panniculitis, oligoarthritis, tuberculosis, erythema nodosum

## Abstract

Erythema nodosum (EN) is a type of panniculitis often benign and self-limited. It may, however, be associated with numerous causes, the most common being infections (namely streptococcal infections), of which tuberculosis (TB) is also one. We report the case of a 43-year-old woman who was referred to our outpatient department with signs of a recurrent EN along with an asymmetrical oligoarthritis of the lower limbs. The investigation excluded all etiological causes of EN, except the interferon gamma release assay (IGRA) which was positive. It was assumed a latent TB infection was the cause of EN. Treatment with isoniazid was started, besides prednisolone and colchicine, with maintained clinical remission. There are just a few cases published about this subject and this intends to emphasize the importance of medical history and of an exhaustive search for a cause, as it may slip away due to the absence of symptoms.

## Introduction

Eryhtema nodosum (EN) is a type of panniculitis (an inflammatory reaction in the subcutaneous adipose tissue) [1,2]. The pathophysiology is not fully understood but the most believed mechanism would be a type IV delayed hypersensitivity response to many antigens [3]. There are potentially many causes associated to EN, the most common being infections (namely streptococcal infections) [1]. Other aetiologies may include drugs, inflammatory bowel disease, sarcoidosis, Beçhet's disease, neoplasia, and pregnancy. From one-third and up to 50% of cases, no cause is found, and it is assumed to be idiopathic [3].

Tuberculosis (TB) is a known cause of EN, despite being less frequent alongside the diminishing of its incidence worldwide [1]. Data show that in 2020, almost 10 million people had an infection with TB; the incidence worldwide is falling at about 2% per year, but this number is far away from the milestone of the End Tuberculosis strategy stated by the World Health Organization [4].

An active TB infection associated with non-septic arthritis is known as Poncet's disease [5]. However, a latent infection may also be responsible for the same clinical picture [6]. We present the case report of a 43-year-old woman with recurrent EN which, after an exhaustive investigation, we assumed to be secondary to a latent TB infection.

## Case presentation

A 43-year-old female, smoker, and with no other relevant medical history, was referred from the emergency room to our rheumatology outpatient department due to a recurrent EN. The patient showed bilateral inflammatory red tender nodules of the anterior aspects of the legs, ranging from bright red to bluish with no ulceration, pitting, or livedo reticularis. It started two weeks before alongside increasing pain and functional disability. No other infections, constitutional symptoms, or risky sexual behavior were reported and no recent travels to endemic countries associated with fungal or mycobacterium infections or insect bites were mentioned. There were no complaints related to rheumatic systemic diseases and the patient denied uveitis. The patient did not take any medication meanwhile. Two similar episodes had happened previously as a self-limited bilateral process with mild pain and no disability: the first occurred nine years before (no cause identified) and the other one four years before during her third pregnancy. Intermittent and self-limited lower limbs paraesthesia (with no clear dermatome) were reported.

On physical examination, subcutaneous, erythematous, and tender nodules in the anterior surface of both legs were observed. No other skin manifestations, including livedo reticularis, were seen and the patient had no arthritis, mucosal changes, or enlarged lymph nodes. The cardiac and pulmonary auscultation was normal, as well as the oropharyngeal examination, and she was apyretic. The patient was also observed by a dermatologist, who did not need a skin biopsy to confirm the diagnosis.

Treatment with nonsteroidal anti-inflammatory drugs (NSAIDs), like acetylsalicylic acid, was started with no clinical relief. One week later she experienced an asymmetrical oligoarthritis of the lower limbs (knees and ankles) and prednisone 40 mg per day (0,6mg/kg/day) was started with a progressive reducing dosage. Despite the symptoms’ relief and medical advice, she abruptly stopped medications because of weight concerns. Musculoskeletal ultrasound examination showed grey scale synovitis (grade 2) with no Doppler signal at the left tibiotalar and subtalar joints, both knees and left posterior tibialis, and right peroneal tendons synovial sheaths. Distal infrapatellar bursitis with significant synovial hypertrophy was observed at both knees. The patient kept treatment with prednisone (20mg per day) alongside colchicine (1mg per day) with partial cutaneous and articular relief. A further investigation was started due to the clinical worsening.

Laboratory evaluation (Table 1) revealed normocytic normochromic anaemia (haemoglobin level 11.2 g/dL; reference range 12-15 g/dL), an elevation of both erythrocyte sedimentation rate (56 mm/h; normal value < 21 mm/h) and C-reactive protein (1.4 mg/dL; normal value < 0.5 mg/dL), with normal kidney function, urinalysis, hepatic profile, iron kinetics, electrophoresis of serum protein, and phosphocalcium metabolite profile. Pregnancy test and throat culture for group A beta-hemolytic streptococcal infection were negative. The assay of immunoglobulin (Ig) A, IgM and IgG were normal. Angiotensin-converting enzyme (ACE) was slightly positive (61 U/L; normal value < 56 U/L) with normal 1,25 hydroxyvitamin D. Immunological study showed a weak positive rheumatoid factor (RF) (17.4 UI/mL; negative if < 15 UI/mL) and antinuclear antibody (with a speckled pattern) with negative extractable nuclear antigen antibodies, antineutrophil cytoplasmic antibodies (ANCA), anti-cyclic citrullinated peptide antibody (anti-CCP), anti-double-stranded DNA (dsDNA) antibodies, antiphospholipid antibodies, cryoglobulins, and normal levels of complement fraction 3 and 4. Both human leukocyte antigens (HLA) B27 and B51 were negative. Bacterial (*Treponema pallidum*, *Coxiella burnetti*, *Brucella *spp, *Bartonella henselae, Borrelia burgdorferi*), viral (cytomegalovirus, Epstein-Barr virus, hepatitis B and C, human immunodeficiency virus), and parasitic (*Toxoplasma gondii*) serologies were negative. The IGRA test (T-SPOT®.TB test; Oxford Immunotec Global Limited, Abingdon, United Kingdom) was positive. Nuclear acid test for severe acute respiratory syndrome coronavirus 2 (SARS-CoV-2) was negative.

**Table 1:** Laboratory results

Parameter	Result	Normal range
Haemoglobin	11.2 g/dL	12-15 g/dL
Erythrocyte sedimentation rate	56 mm/h	< 21 mm/h
C-reactive protein	1.4 mg/dL	< 0.5 mg/dL
Creatinine	0.54 mg/dL	0.47 - 1.04 mg/dL
Aspartate transaminase	40 U/L	7 – 52 U/L
Alanine transaminase	35 U/L	8 - 48 U/L
Angiotensin-converting enzyme	61 U/L	< 56 U/L
Rheumatoid factor	17.4 UI/mL	< 15 UI/mL

Abdominal and pelvic computed tomography (CT) scans were normal and thoracic CT scan excluded active TB, mediastinal and hilar lymphadenopathy, and tumour diseases, revealing lung parenchyma with fibrotic sequelae in the middle lobe, lingula, and basis with some bilaterally ground-glass opacification, with no granulomas or enlarged lymph nodes. Further multidisciplinary meetings with radiology and pneumology departments excluded active diseases. Complete axial and peripheric articular X-ray exams did not show bone erosions at the intra-articular, periarticular or enthesopathic points, or enthesopathies. Thyroid ultrasound exam and lower limbs electromyographic exam were normal. The gynaecological report by her doctor excluded active infections and confirmed normal investigation follow-up procedures.

Initially, the patient was prescribed aspirin with no effect. Then prednisone 40mg per day was prescribed with a good response. After a sudden interruption of the medication, which resulted in a cutaneous and articular and periarticular synovitis flare, she restarted prednisone 20mg per day (following a weaning scheme) and colchicine 1mg per day with a progressive cutaneous and musculoskeletal remission. The addition of an empiric trial of doxycycline in a seven-day regimen did not help, as could be expected of a bacterial reactive process.

After all the investigation results and considering the exclusive positive IGRA exam and an articular involvement typical of reactive arthritis, she was started on isoniazid (300mg per day) and pyridoxine (20mg per day) with good tolerance.

**Figure 1:**
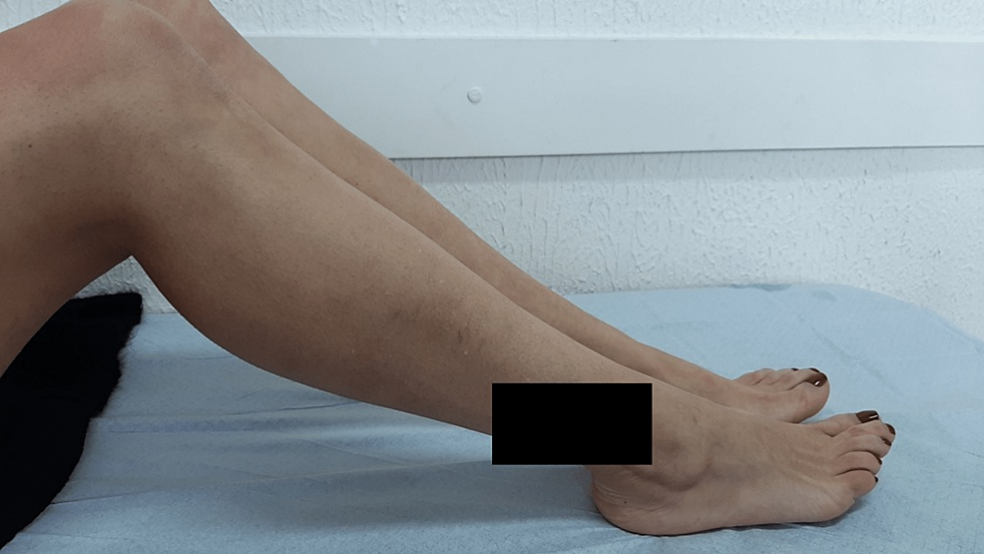
Lateral perspective of the legs of the patient after initiating isoniazid (four-month follow-up period) Note: no photographs are available before the beginning of the treatment

After four weeks of treatment with prednisone and colchicine, there was a significant improvement in symptoms (Figure 1). Prednisone dose was progressively diminished until its suspension. The patient kept treatment with isoniazid, with good tolerance, with the goal of achieving nine months of therapy. No more EN nor arthritis episodes were observed during the 12-month follow-up period.

## Discussion

This case aroused many differential diagnoses considering the multivariable nature of EN. Lofgren syndrome is a type of acute and benign sarcoidosis characterized by EN, bilateral hilar lymphadenopathy, and polyarthritis [7]. Fever and uveitis may also be present. In this case, although the patient experienced arthritis and EN, with a discrete elevated ACE, no calcium or vitamin D changes were observed, as no lymphadenopathy was seen in the CT scan. This makes the diagnosis unlikely. Studies also show that ACE levels have poor sensitivity (60%) and specificity (70%) for the diagnosis of sarcoidosis and many other factors may increase its levels [8].

Cutaneous and articular symptoms were not supported by typical immunological antibodies or clinical findings: absence of livedo reticularis and neurological signs (suggestive of polyarteritis nodosa); negative ANCA antibodies (typical of ANCA vasculitis); no symmetrical additive polyarthritis with positive RF and anti-CCP, accompanied by X-ray articular erosions (indicative of rheumatoid arthritis); absence of mucous ulcers and no detection of HLA-B51 (Behçet's disease).

Pregnancy, active respiratory, abdominal, and urogenital infections were excluded, as well as a paraneoplastic syndrome. There were no symptoms of inflammatory bowel disease or previous putative pharmacological interventions.

Besides recurrent bilateral lower limb EN, the patient experienced asymmetric oligoarthritis of the lower limbs and bilateral tenosynovitis and the ultrasound findings were similar to the ones found in spondylarthritis, like reactive arthritis, although no axial involvement was observed and HLA-B27 was negative. The only positive investigation exam was the IGRA blood analysis. Therefore, we assumed reactive arthritis and EN secondary to latent TB.

This case reports a recurrent EN with concomitant evolution to an asymmetrical oligoarthritis of the lower limbs, with no erosive or destructive course, whose sustained clinical remission was achieved after isoniazid treatment of a TB latent infection.

EN is considered a hypersensitivity reaction to certain stimuli, with the formation and deposition of immunocomplexes in the subcutaneous fat tissue, affecting the connective tissue septa or the lobules of fat themselves [2].^ ^There is a neutrophilic infiltration with the formation of reactive oxygen species [9]. Therefore, external stimuli that may have a similar pathophysiology may be coincident. It is often a benign condition that usually solves spontaneously or with symptomatic treatment using NSAIDs. If the identified underlying cause is solved, refractory symptoms may be treated with corticosteroids [1,3]. Here, the evolution to an oligoarthritis similar to spondylarthritis (like reactive arthritis) justified the investigation of infectious agents behind these symptoms, with TB being the only finding.

The diminishing prevalence of TB in developed countries makes this diagnosis a rare cause of EN; however, as it is still a common disease in developing countries, it should be considered as part of the differential diagnosis [4,5]. Aseptic polyarthritis associated with active TB is known as Poncet's disease and behaves as reactive arthritis, typically non-destructive, non-erosive, and with a fast response to anti-TB treatment [5]. TB arthritis, diagnosed after *Mycobacterium tuberculosis* in the synovial fluid, acts as a fast erosive and destructive arthritis and usually shows a monoarticular involvement with slow inflammatory relief during the long-standing therapy.

Latent TB infection can be diagnosed with a skin test, Mantoux tuberculin skin test (TST) (the result of which can be false with previous bacille Calmette-Guérin (BCG) vaccine or due to immunosuppressive conditions), and the blood IGRA test (with two available diagnostic methods: QuantiFERON-Plus (QIAGEN N.V., Venlo, The Netherlands), and T-Spot) [10]. In our hospital, the TST are no longer used, and the TB blood tests are the most implemented ones.

In this case, the patient had a positive IGRA (T-Spot) but presented no night sweats, weight loss, anorexia, fever, or fatigue. The CT scan showed no suggestive images of active pulmonary TB disease. Therefore, latent TB was assumed, and considering that the patient was under corticosteroids (cumulative prednisone dose superior to 1500mg), the decision to treat was made after a discussion with the infectious diseases department and the patient started the therapy with isoniazid.

Most of the case reports published about EN and TB infection are about active infection. There are few cases associated with latent TB infection, usually concerning younger patients (up to 18 years with complete resolution of the symptoms after treatment) [11,12].

## Conclusions

This case report aims to highlight TB as an important cause to be investigated in the face of a case of EN and a reactive pattern of oligoarthritis, even in the adult population. One should keep in mind that EN may have multiple aetiologies, so a thorough investigation is essential to the diagnosis. TB is still a prevalent infectious disease, and we should always consider it in our differential diagnosis, especially if an obvious clinical reason is not evident. A multidisciplinary approach is essential in the diagnosis and follow-up of these patients.
